# The Impact of COVID-19 on Behavior and Physical and Mental Health of Romanian College Students

**DOI:** 10.3390/medicina58020246

**Published:** 2022-02-06

**Authors:** Sînziana Călina Silișteanu, Maria Totan, Oana Raluca Antonescu, Lavinia Duică, Elisabeta Antonescu, Andrei Emanuel Silișteanu

**Affiliations:** 1 Faculty of Medicine and Biological Sciences, “Stefan cel Mare” University of Suceava, 13 Universitatii Str., 720229 Suceava, Romania; sinziana.silisteanu@usm.ro; 2 Faculty of Medicine, “Lucian Blaga” University of Sibiu, 2A Lucian Blaga Str., 550169 Sibiu, Romania; lavinia.duica@ulbsibiu.ro (L.D.); elisabeta.antonescu@ulbsibiu.ro (E.A.); 3 County Clinical Emergency Hospital, 2-4 Corneliu Coposu Str., 550245 Sibiu, Romania; 4 Faculty of Political, Administrative and Communication Sciences of Cluj-Napoca, 71 Traian Mosoiu Street, 400132 Cluj Napoca, Romania; silisteanuandrei10@yahoo.com

**Keywords:** students, psycho-emotional moods, behavioral problems, academic performance, COVID-19 pandemic

## Abstract

*Background and Objectives*: The COVID-19 pandemic caused by SARS-CoV-2 significantly marked people’s lives with respect to their behavior, and their physical and mental health. *Materials and Methods*: This is a cross-sectional study that was conducted in 2021 for a period of 5 months. The study sample included 218 students from the College of Physical Education and Sports of the University of Suceava who filled in a questionnaire on mental, physical and behavioral symptoms caused by the COVID-19 pandemic, as well as the Anxiety Assessment Questionnaire (STAI). *Results*: The responses indicated increased anxiety, physical symptoms, altered behavior, and increased perception of social restrictions. Regression analyses indicated that the levels of anxiety during the COVID-19 outbreak were strongly correlated with cognitive, physical and behavioral symptoms of the students. These were influenced by the living arrangements, location (urban vs. rural), age group and study year. *Conclusions*: The results show that first-year students did not exhibit significant physical and cognitive symptoms despite reporting anxiety, probably due to their enthusiasm as beginners. The 3rd year students were prone to anxiety and reported cognitive symptoms, possibly due to the prospects of an uncertain future.

## 1. Introduction

The COVID-19 pandemic caused by the SARS-CoV-2 marked people’s lives significantly by influencing their behavior, but also their physical and mental health. The World Health Organization issued specific guidelines and urged an analysis on the effects of the pandemic on mental health and possible psycho-social consequences [1]. Social isolation, restrictions on physical and social contact, fear of illness and the loss of loved ones are just some of the aspects caused by the pandemic [2,3]. It has been well documented that quarantine and self-isolation can affect the daily activities of the population by worsening their anxiety, depression, loneliness, insomnia and development of behavior that can lead to increased alcohol consumption, tobacco or substance use, and even suicide [4].

Previous studies have consistently shown the psychosocial impact of the COVID-19 pandemic on mental health and in particular on women and children of young age [3,5,6]. For example, one study [7] showed that approximately 1.5 billion young people, of which 90% were students enrolled in schools worldwide, did not attend classes. In China, more than 220 million children and adolescents stayed at home during long periods of times because of the pandemic [8], and 18.6% showed symptoms characteristic of anxiety disorders [9]. Thus, Chinese students were found to be at higher risks in comparison to the adult population in terms of anxiety, stress or depression [3] and were very sensitive to the negative effects of the quarantine [10,11,12].

The unprecedented situation created by the COVID-19 pandemic in Romania has transformed the educational system by forcing teachers as well as students to adapt within a very short time to the new social conditions and to the online learning process [13,14,15]. Suceava county, situated in the north-east region of Romania, saw the first and the largest COVID-19 outbreak that originated at Suceava regional county hospital. As a consequence, Suceava county was placed under complete lockdown, which had a major impact on all ages, including young individuals. Being the first region in the country under sheltering-in-place measures, students in this area were also the first ones subject to the social restrictions and sudden changes in the curriculum delivery. Therefore, we hypothesized that these measures had a negative impact on students’ health due to increased stress and anxiety since there was no history on how to successfully cope with such sudden and adverse environmental factors [16]. To address this, we evaluated the impact of the first COVID-19 outbreak on physical, mental and behavioral manifestations of first-, second- and third-year college students under complete lockdown conditions in the county of Suceava, Romania.

## 2. Materials and Methods

The study was cross-sectional and was conducted in 2021 for a period of 5 months at “Ștefan cel Mare” University of Suceava.

The target population was represented by first-, second- and third-year students from the College of Physical Education and Sports at “Ștefan cel Mare” University in Suceava. Off the 412 students contacted, 242 responded to our online questionnaire, and 218 actually completed the questionnaires. All participants received the information package about the study and signed informed consent. The study was conducted online, and students received one questionnaire as well as the self-evaluation STAI (State-Trait Anxiety Inventory Spielberger) questionnaire, via e-mail, using Google Docs.

The study was approved by the Research Ethics Committee of the University of Suceava nr. 34/20.05.2021, in compliance with the ethical principles on human medical research according to the Declaration of Helsinki. The collected data were anonymous and confidential, and were used only for this study.

The general questionnaire was designed to evaluate the impact of the COVID-19 pandemic during the lockdown/isolation period on the emotional, cognitive and physical status of the students and also its influence on their quality of life. The questions were closed type, with multiple answer choices. The students had to choose from the options provided, with the used terms being easy to understand without creating confusion. The questions were short and precise, meant to elicit a direct answer and to be easily processed for analyses. The questions focused on mental reactions (emotional and cognitive) and physical and behavioral reactions reported by students in response to the lockdown (isolation, online courses) by assessing stress, fear, anxiety and panic. For cognitive evaluation, the questionnaire aimed to determine whether or not the student had difficulties in concentration/attention and coping with catastrophic events. For physical evaluation, the symptoms presented and recorded were headache, fatigue, myalgia, anorexia and malaise. The questionnaire also included questions that assessed changes in the general behavior of the students, such as eating, sleep, alcohol consumption, tobacco use, physical activity and social restrictions.

Students also completed the STAI questionnaire that included 40 items, assessed on a 4-point Likert scale (e.g., from “Almost Never” to “Almost Always”). The scale has 2 subscales: one for the state, with 20 items that reflects the person’s condition “at this moment” and the trait subscale, also with 20 items, that reflects the person’s condition “in general” [17]. This questionnaire had been used and validated in previous Romanian studies, albeit in other models [18]. It has been often used in assessing anxiety and is a sensitive predictor of distress. State anxiety items include: “I am tense; I am worried” and “I feel calm; I feel secure.” The possible scores vary from a minimum of 20 points (signs are not present at all) to a maximum of 80 points (signs are very present). Trait anxiety items include: “I worry too much over something that really doesn’t matter” and “I am content; I am a steady person”. Likewise, the possible scores vary from a minimum of 20 points (signs are not present at all) to a maximum of 80 points (signs are very present). Data were analyzed with Student’s *t*-test and regression analysis using Microsoft Excel and IBM SPSS Statistics for Windows version 24.0 (Armonk, NY: IBM Corp) statistical software. Data are presented as absolute numerical values, mean (±SD) as well as proportions. Values with *p* < 0.05 were considered statistically significant.

## 3. Results

Of the 218 students who completed the questionnaires, 155 (71.11%) were female and 63 (28.89%) were male. The proportion by study years was 75 (34.40%), 72 (33.03%) and 71 (32.57%), for the first, second and third year, respectively (Table 1).

Our analyses showed that anxiety due to stress, fear, and panic attacks were observed more in females than in males (57.79% vs. 19.72%) while 72.93% of students reported difficulty in concentration/attention. A similarly high percentage of students complained of various physical symptoms, such as fatigue, myalgia, headache, malaise and anorexia. Additionally, prolonged sitting position at the computer caused pain in the lumbar and dorsal spine in a greater proportion than visual acuity disorders and low limb pain (Table 2).

A high number of students reported increased consumption of carbohydrates (37.15%) and fats (31.65%), tobacco use (32.11%) and coffee (28.89%) during lockdown. The number of students with sleeping difficulty (was quite similar with those who slept well (29.35% vs. 31.19%). Students perceived the restrictions imposed by the COVID-19 pandemic more in terms of social restrictions than physical restriction (38.99% vs. 30.73%).

When comparing students’ responses by the study year, there were significant differences within each age group and between study year for most emotional, cognitive, behavioral, and physical manifestations. However, with very few exceptions, the physical activity was not significantly impacted by the lockdown (Table 3).

When examining the effect of living arrangements on students’ symptoms, again there are significant differences between each study year for most categories. However, there were no significant differences in physical symptoms between first and second year for students living in the dorm, nor there were any effects on physical activity for students living alone or with their families. Similarly, there were no significant effects on physical activity between first and third year for all living arrangements (Table 4). When examining the effects of urban *versus* rural, it seems that urban environment had a greater impact on all symptom categories compared to rural area. Again, the least affected was the physical activity between study year (Table 4).

Anxiety scores using STAI for state anxiety were 50.08, 49.28 and 49.66 for first, second, and third year, respectively, and for trait anxiety they were 49.97, 48.65 and 47.23 for first, second, and third year, respectively. This indicates a moderate-to-high anxiety level in the students. Linear regression analyses show a significant enhancement of the affective and physical symptoms for the 26–30 age category, of cognitive symptoms for the 21–25 age category and of physical activity for the 18–20 age category (Table 5).

Regression analyses also revealed significant differences between urban and rural environments. As such, the urban environment had a significant effect on cognitive symptoms, while the rural environment affected affective, cognitive, behavioral and physical symptoms as well as STAI trait anxiety (Table 6).

Finally, compared to students who lived with the family or alone, more students who lived in the dorm reported affective, cognitive and behavior symptoms as well as a significant increase in trait anxiety (Table 7).

## 4. Discussions

This study was conducted to determine the extent to which the lockdown during the first and largest COVID-19 outbreak in the county of Suceava, Romania, had on psychological, physical and behavioral manifestations of first-, second- and third-year college students. Overall, our findings show a significant increase in anxiety, as measured by STAI, as well as in reporting affective, cognitive, behavioral and physical symptoms by the students following physical restriction, quarantine and self-isolation.

The negative health-related conditions caused by the COVID-19 pandemic coupled with the public measures taken to slow the spread of the virus resulted in significant lifestyle changes in the majority of the population. Adaptability to changes was reflected by an increase in stress levels of many individuals that can trigger emotional, behavioral and physiological responses. Within the student population, anxiety has significantly increased primarily due to the fear of being infected and by the cancellation of on-site academic activities. In other words, the individual emotional reactions were caused, on one hand, by the restrictions imposed due to the COVID-19 pandemic and, on the other hand, by exposure to a demanding, competitive environment, without direct physical and social participation [19]. In our study, anxiety, under many forms was encountered in 73.39% of the total number of students. This is in line with the study of Wang et al. [20] who found that the most common emotional response of people during the pandemic was anxiety. Another study also found that the prevalence of anxiety among college students during this pandemic was 27% [6].

During the COVID-19 pandemic, the mental health of university healthcare students was affected negatively. For example, the Hospital Anxiety and Depression Scale (HADS) revealed that only 43.8% and 40.0% of participants had normal anxiety and depression scores, while 22.4% showed borderline abnormal anxiety/depression scores, with many students (33.8%) being identified with abnormal anxiety scores [21]. Among these psychological effects, other studies showed intense stress, irritability, anxiety, fear, complaints of depression and post-traumatic stress disorder as well as sleep disorders [10,22].

Besides changes in affectivity, our students reported cognitive alterations, such as difficulties in concentration (72.93%) and catastrophic thinking (25.22%). Intolerance of uncertainty includes the belief that uncertain events are unfair, unacceptable and threatening [23]. In such situations, catastrophic cognitions occur, which mediate the relationship between information seeking and health anxiety [24]. It was also found that catastrophic thinking about COVID-19 can contribute to various psychiatric symptoms associated with depression, agoraphobia and panic disorder [25].

We also assessed several psychosomatic symptoms in students during lockdown such as fatigue, anorexia, myalgia, headache, and malaise, which were reported by over 20% of the students. COVID-19 threat negatively affected the biological rhythm through intolerance of ambiguity, which then increases somatic symptoms both directly and through biological rhythm. A review of 13 articles showed that the COVID-19 pandemic had a negative impact on physical and mental health in healthcare workers, and headaches have been associated to psychological stress and work overload during the pandemic [26].

Other behaviors, such as sleep, eating, daily activities (sports) and social activities, have all been impacted or restricted due to changes in working conditions, and enforcement of quarantine or curfews. In our study, 50% of students reported at least one unhealthy habit, such as alcohol consumption and tobacco use or insomnia. In the United Kingdom, the alcohol consumption increased by 4.5% increase during April to October 2020, compared to the same period in the previous year [27]. In a study involving students, however, there was a reduction in alcohol drinking during COVID-19 pandemic. Thus, living with parents during emerging adulthood may be protective against heavy drinking [28]. A large study (*n* = 3396) on current tobacco users showed a 28% increase in cigarette use during the pandemic, while 15% reported a decrease in their tobacco use. The most common reasons for increased use were increased stress, more time at home, and boredom while quarantined, while the most common reasons for reduced use were health concerns and more time around non-smokers [29]. Students decreased smoking and vaping frequency from the week prior to their campus closing; however, decreased frequency did not correspond to reduced quantity. Higher anxiety and moving home (versus living independently) were related to the decrease [30].

Our study showed that students increase their food consumption. Some studies showed a marked increase in body mass index during the quarantine [31]; however, in a study conducted in Serbia, most students did not feel a constant need for food (63.2%), nor did they consume larger amounts of food than usual (67.5%). Students (36.0%) were careful about the nutritional and energy value of food, and they had well-balanced meals that had a beneficial effect on their immune responses [32].

Our students were more affected by the social restrictions (38.99%) than the physical restrictions (30.73%), which is in line with other studies showing student dissatisfaction with social restrictions [33]. Physical symptoms were also present in this study to a great extent, with half of students reporting dorsal and lumbar pain. The low back pain (LBP) was the most common musculoskeletal pain area that increased after the quarantine. LBP was a highly prevalent health problem in medical students in Serbia, too [34]. In other recent studies conducted in Saudi Arabia, the point prevalence of LBP found was 40.5% in medical students [35], 21.2% among health sciences students [36], in comparison with 80% in nurses [37], and 31.4% in office workers [38]. This could be related to the burden of work, type of professional or academic activity carried out by each group, and poor posture at work [39].

Regression analyses indicated that the levels of anxiety during the COVID-19 outbreak were strongly correlated with cognitive, physical and behavioral symptoms of the students. These were influenced by the living arrangements, location (urban vs. rural) and age group. Our study has several limitations that include a relatively small number of participants as well as the use of the general questionnaire that has not been internally validated. The fact that the questionnaires used to measure human feelings and emotions are self-reported is another limitation of this study. Notwithstanding this, our study reveals a significant impact on students’ affective, cognitive, behavioral and physical symptoms following the first largest COVID-19 outbreak in Suceava county, Romania.

## 5. Conclusions

The impact of the COVID-19 pandemic on the students’ physical and mental state resulted in a state of anxiety coupled with a complex of physical, emotional, cognitive and behavioral symptoms. The results also show that first-year students showed less anxiety, perhaps due to their enthusiasm, while third-year students were more prone to anxiety and experienced cognitive symptoms to a greater extent, possibly due to the prospects of an uncertain future, such as final exams and starting a career.

## Figures and Tables

**Table 1:** Socio-demographic characteristics of the study group.

Students		1st Year		2nd Year		3rd Year	
		F	M	F	M	F	M
		59 (78.67%)	16 (21.33%)	51 (70.83%)	21 (29.17%)	45 (63.38%)	26 (36.62%)
Age	<20 years	17	4	11	8	-	-
	21–25 years	22	6	14	3	15	7
	26–30 years	9	2	12	5	16	10
	31–35 years	8	3	8	3	8	5
	>36 years	3	1	6	2	6	4
Location	University dorms	25	8	20	7	21	7
	Living with family	19	4	15	8	14	8
	Living alone	15	4	16	6	10	11
Geographical Area	Urban	40	10	21	11	22	16
	Rural	19	6	30	10	23	10

**Table 2:** Results from the questionnaire on mental, physical and behavioral symptoms caused by COVID-19 pandemic.

Question	Answer	1st Year (*n*,%)		2nd Year (*n*,%)		3rd Year (*n*,%)	
		Female	Male	Female	Male	Female	Male
What was your first reaction when you found out from mass media about the COVID 19 infection?	Fear	20 (26.67)	3 (4)	8 (11.11)	5 (6.94)	12 (16.91)	5 (7.04)
	Frustration	15 (20)	5 (6.67)	6 (8.33)	3 (4.16)	12 (16.91)	8 (11.27)
	Panic attack	8 (10.6)	3 (4)	20 (27.78)	6 (8.33)	12 (16.91)	5 (7.04)
	Stress	14 (18.6)	4 (5.33)	15 (20.83)	6 (8.33)	7 (9.86)	6 (8.45)
	I don’t know	2 (2.67)	1 (1.33)	2 (2.78)	1 (1.38)	2 (2.82)	2 (2.82)
What symptoms did you experience when classes were moved online?	Fatigue	11 (14.67)	2 (2.67)	14 (19.44)	4 (5.56)	8 (11.27)	4 (5.63)
	Anorexia	12 (16)	2 (2.67)	10 (13.88)	2 (2.78)	8 (11.27)	3 (4.23)
	Myalgia	14 (18.67)	4 (5.33)	13 (18.05)	5 (6.94)	4 (5.63)	7 (9.86)
	Headache	10 (1.33)	4 (5.33)	6 (8.33)	6 (8.33)	8 (11.27)	4 (5.63)
	Malaise	12 (16)	4 (5.33)	8 (11.11)	4 (5.56)	7 (9.86)	8 (11.27)
Did lockdown cause the appearance or worsening of cognitive/attention disorders?	Difficulty in concentration	27 (36)	7 (9.33)	16 (22.22)	7 (9.72)	18 (25.35)	7 (9.86)
	Difficulty in attention	21 (28)	4 (5.33)	24 (33.33)	8 (11.11)	12 (16.91)	8 (11.27)
	Catastrophic events	10 (13.33)	4 (5.33)	10 (13.89)	6 (8.33)	15 (21.13)	10 (14.08)
	I don’t know	1 (1.33)	1 (1.33)	1 (1.38)	0	0	1 (1.41)
Which food categories did you eat more often during lockdown?	Fats	16 (21.33)	4 (5.33)	23 (31.94)	6 (8.33)	12 (16.91)	8 (11.27)
	Proteins	13 (17.33)	6 (8)	12 (16.67)	8 (11.11)	20 (28.17)	7 (9.86)
	Carbohydrates	30 (40)	6 (8)	14 (19.44)	7 (9.72)	13 (18.31)	11 (15.49)
Describe the quality of your sleep during the lockdown	Sleep difficulty	16 (21.33)	4 (5.33)	20 (27.78)	6 (8.33)	12 (16.91)	6 (8.45)
	Insomnia	12 (16)	5 (6.67)	6 (8.33)	6 (8.33)	12 (16.91)	9 (12.68)
	Deep sleep	22 (29.33)	3 (4)	20 (27.78)	5 (6.94)	12 (16.91)	6 (8.45)
	No change	5 (6.67)	4 (5.33)	3 (4.17)	3 (4.17)	9 (12.68)	5 (7.04)
	I don’t know	4 (5.33)	0	2 (2.78)	1 (1.38)	0	0
How did you perceive the restrictions during the lockdown?	Physical restriction	14 (18.67)	5 (6.67)	16 (22.22)	8 (11.11)	16 (22.54)	8 (11.27)
	Social restriction	25 (33.33)	8 (10.67)	20 (27.78)	6 (8.33)	18 (25.35)	8 (11.27)
	Imposed self-isolation	20 (26.67)	3 (4)	14 (19.44)	6 (8.33)	9 (12.68)	6 (8.45)
	I don’t know	0	0	1 (1.38)	1 (1.38)	2 (2.82)	2 (2.82)
Was your alcohol/tobacco/coffee consumption influenced by the lockdown?	Tobacco	15 (20)	8 (10.67)	10 (13.89)	8 (11.11)	14 (19.72)	15 (21.13)
	Alcohol	2 (2.67)	6 (8)	3 (4.17)	5 (6.94)	3 (4.23)	6 (8.45)
	Tobacco and alcohol	10 (13.33)	5 (6.67)	3 (4.17)	3 (4.17)	3 (4.23)	6 (8.45)
	Coffee	12 (16)	4 (5.33)	14 (19.44)	7 (9.72)	16 (22.54)	10 (14.08)
What symptoms did you experience during online education?	Cervical spine pain	14 (18.67)	4 (5.33)	12 (16.67)	3 (4.17)	11 (15.49)	4 (5.63)
	Dorsal spine pain	18 (24)	5 (6.67)	10 (13.89)	6 (8.33)	15 (21.13)	3 (4.23)
	Lumbar spine pain	13 (17.33)	5 (6.67)	16 (22.22)	10 (13.89)	12 (16.91)	8 (11.27)
	Visual acuity disorders	6 (8)	2 (2.67)	7 (9.72)	1 (1.38)	4 (5.63)	5 (7.04)
	Low limb pain	8 (10.67)	0	6 (8.33)	1 (1.38)	3 (4.23)	6 (8.45)

**Table 3:** Effects of age group on students’ symptoms between study years (*t*-test).

Signs	Age	1st Year/2nd Year	2nd Year/3rd Year	1st Year/3rd Year
		*p*	*p*	*p*
Affective symptoms	<20 years	0.0171	-	-
	21–25 years	0.0026	0.0565	0.0541
	26–30 years	0.0115	0.0492	0.0601
	31–35 years	0.0487	0.0465	0.0023
	>36 years	0.1062	0.0496	0.0631
Cognitive symptoms	<20 years	0.0328	-	-
	21–25 years	0.0192	0.0049	0.0241
	26–30 years	0.0571	0.0081	0.0495
	31–35 years	0.0419	0.0238	0.0185
	>36 years	0.0172	0.0764	0.0607
Behavioral symptoms	<20 years	0.0086	-	-
	21–25 years	0.0688	0.0619	0.0076
	26–30 years	0.0527	0.0866	0.0372
	31–35 years	0.0003	0.0143	0.0141
	>36 years	0.0364	0.0039	0.0402
Physical symptoms	<20 years	0.0021	-	-
	21–25 years	0.0269	0.0052	0.0219
	26–30 years	0.0407	0.0053	0.0459
	31–35 years	0.0015	0.0858	0.0845
	>36 years	0.1061	0.0163	0.0929
Physical activity	<20 years	0.2721	-	-
	21–25 years	0.2370	0.2172	0.2217
	26–30 years	0.3102	0.0069	0.3128
	31–35 years	0.2965	0.0012	0.2965
	>36 years	0.0758	0.0824	0.1417

**Table 4:** Effects of living arrangements and geographical area on students’ symptoms between study year during lockdown (*t*-test).

Signs	Living Arrangements				Geographical Area			
		1st Year/ 2nd Year	2nd Year/ 3rd Year	1st Year/ 3rd Year		1st Year/ 2nd Year	2nd Year/ 3rd Year	1st Year/ 3rd Year
		*p*	*p*	*p*		*p*	*p*	*p*
Affective symptoms	*Living in dorms*	0.0318	0.0264	0.0055	*Urban*	0.0184	0.0428	0.0248
	*Living with family*	0.0284	0.0262	0.0022	*Rural*	0.0114	0.0052	0.0166
	*Living alone*	0.0108	0.0396	0.0499				
Cognitive symptoms	*Living in dorms*	0.0196	0.0471	0.0281	*Urban*	0.0021	0.0321	0.0341
	*Living with family*	0.0407	0.0044	0.0365	*Rural*	0.0017	0.0333	0.0349
	*Living alone*	0.0474	0.0111	0.0367				
Behavioral symptoms	*Living in dorms*	0.0325	0.0346	0.0022	*Urban*	0.0206	0.0267	0.0062
	*Living with family*	0.0520	0.0304	0.0223	*Rural*	0.3578	0.0261	0.0098
	*Living alone*	0.0188	0.0351	0.0165				
Physical symptoms	*Living in dorms*	0.1781	0.0025	0.1796	*Urban*	0.0057	0.0044	0.0013
	*Living with family*	0.0016	0.0468	0.0483	*Rural*	0.0298	0.0361	0.0645
	*Living alone*	0.0462	0.0256	0.0212				
Physical activity	*Living in dorms*	0.0113	0.2096	0.2067	*Urban*	0.0409	0.0013	0.0422
	*Living with family*	0.2771	0.0072	0.2759	*Rural*	0.0802	0.1786	0.2074
	*Living alone*	0.4261	0.0027	0.4237				

**Table 5:** Effects of age on STAI and students’ symptoms.

Age (Years)	STAI (State Anxiety)		STAI (Trait Anxiety)		Affective Symptoms		Cognitive Symptoms		Behavioral Symptoms		Physical Symptoms		Physical Activity	
	F	P	F	P	F	P	F	P	F	P	F	P	F	P
18–20	0.006	0.939	0.164	0.688	0.250	0.620	0.16	0.900	2.130	0.153	0.472	0.496	8.184	0.007
21–25	0.162	0.689	0.418	0.520	3.522	0.065	5.045	0.028	2.576	0.113	0.022	0.884	0.107	0.744
26–30	0.116	0.735	1.712	0.196	29.049	0.000	0.530	0.470	0.665	0.418	7.375	0.009	0.937	0.337
31–35	0.247	0.623	0.004	0.950	0.571	0.456	0.067	0.798	0.207	0.652	1.098	0.303	0.002	0.968
>36	0.924	0.348	0.567	0.460	0.342	0.565	0.024	0.878	2.276	0.147	2.861	0.106	3.460	0.078

**Table 6:** Effects of geographical area on STAI and students’ symptoms.

Geographical Area	STAI (State Anxiety)		STAI (Trait Anxiety)		Affective Symptoms		Cognitive Symptoms		Behavioral Symptoms		Physical Symptoms		Physical Activity	
	F	P	F	P	F	P	F	P	F	P	F	P	F	P
Urban	0.004	0.950	0.586	0.446	0.003	0.957	23.454	0.000	3.241	0.074	0.791	0.376	0.633	0.428
Rural	0.004	0.951	5.994	0.016	6.651	0.011	11.003	0.001	6.570	0.012	12.258	0.0001	0.963	0.328

**Table 7:** Effects of living arrangements on STAI and students’ symptoms.

Living Arrangements	STAI (State Anxiety)		STAI (Trait Anxiety)		Affective Symptoms		Cognitive Symptoms		Behavioral Symptoms		Physical Symptoms		Physical Activity	
	F	P	F	P	F	P	F	P	F	P	F	P	F	P
Living in dorms	0.095	0.759	7.061	0.009	4.967	0.028	9.545	0.003	9.573	0.003	0.461	0.499	3.877	0.052
Living with family	0.126	0.724	0.002	0.962	1.567	0.215	15.371	0.000	3.825	0.055	0.141	0.709	0.475	0.493
Living alone	0.652	0.422	1.224	0.273	0.861	0.357	9.658	0.003	2.236	0.140	0.921	0.341	2.418	0.125

## Data Availability

Data are contained within the article.
